# Can Antioxidants Protect Against Disuse Muscle Atrophy?

**DOI:** 10.1007/s40279-014-0255-x

**Published:** 2014-10-30

**Authors:** Scott K. Powers

**Affiliations:** Department of Applied Physiology and Kinesiology, University of Florida, PO Box 118205, Gainesville, FL 32611 USA

## Abstract

Long periods of skeletal muscle inactivity (e.g. prolonged bed rest or limb immobilization) results in a loss of muscle protein and fibre atrophy. This disuse-induced muscle atrophy is due to both a decrease in protein synthesis and increased protein breakdown. Although numerous factors contribute to the regulation of the rates of protein breakdown and synthesis in skeletal muscle, it has been established that prolonged muscle inactivity results in increased radical production in the inactive muscle fibres. Further, this increase in radical production plays an important role in the regulation of redox-sensitive signalling pathways that regulate both protein synthesis and proteolysis in skeletal muscle. Indeed, it was suggested over 20 years ago that antioxidant supplementation has the potential to protect skeletal muscles against inactivity-induced fibre atrophy. Since this original proposal, experimental evidence has implied that a few compounds with antioxidant properties are capable of delaying inactivity-induced muscle atrophy. The objective of this review is to discuss the role that radicals play in the regulation of inactivity-induced skeletal muscle atrophy and to provide an analysis of the recent literature indicating that specific antioxidants have the potential to defer disuse muscle atrophy.

## Introduction

Skeletal muscles are essential for both breathing and locomotion in humans and other animals. Prolonged periods of skeletal muscle disuse (e.g. chronic bed rest, limb immobilization or space flight) can lead to muscle atrophy, impaired contractile performance and overall muscle weakness. The establishment of a countermeasure to prevent disuse muscle atrophy requires a mechanistic understanding of the cellular signalling pathways that regulate both protein synthesis and protein breakdown in muscle. In this regard, ongoing research in muscle biology has improved our understanding of those factors that contribute to inactivity-induced muscle atrophy, and evidence indicates that disturbed redox signalling, due to increased production of reactive oxygen species (ROS) and decreased antioxidant capacity, is an important regulator of signalling pathways that control both proteolysis and protein synthesis in skeletal muscle [1–8]. These collective results are consistent with the concept that oxidative stress plays an important regulatory role in disuse skeletal muscle atrophy, and they raise the question as to whether antioxidant supplementation is a potential countermeasure to protect against inactivity-induced muscle atrophy.

The objectives of this review are twofold: (1) to summarize the current knowledge regarding the mechanistic links between ROS and muscle atrophy resulting from prolonged periods of contractile inactivity; and (2) to discuss the evidence indicating that antioxidant supplementation can protect skeletal muscles against disuse muscle atrophy. The discussion begins with a summary of the experimental models that are used to investigate disuse muscle atrophy. This is followed by an overview of the signalling pathways connecting ROS to decreased protein synthesis and increased proteolysis in skeletal muscle fibres. The review concludes with a summary of the evidence supporting the concept that specific antioxidant compounds can delay disuse muscle atrophy.

## Disuse Muscle Atrophy: Experimental Models

**Fig. 1:**
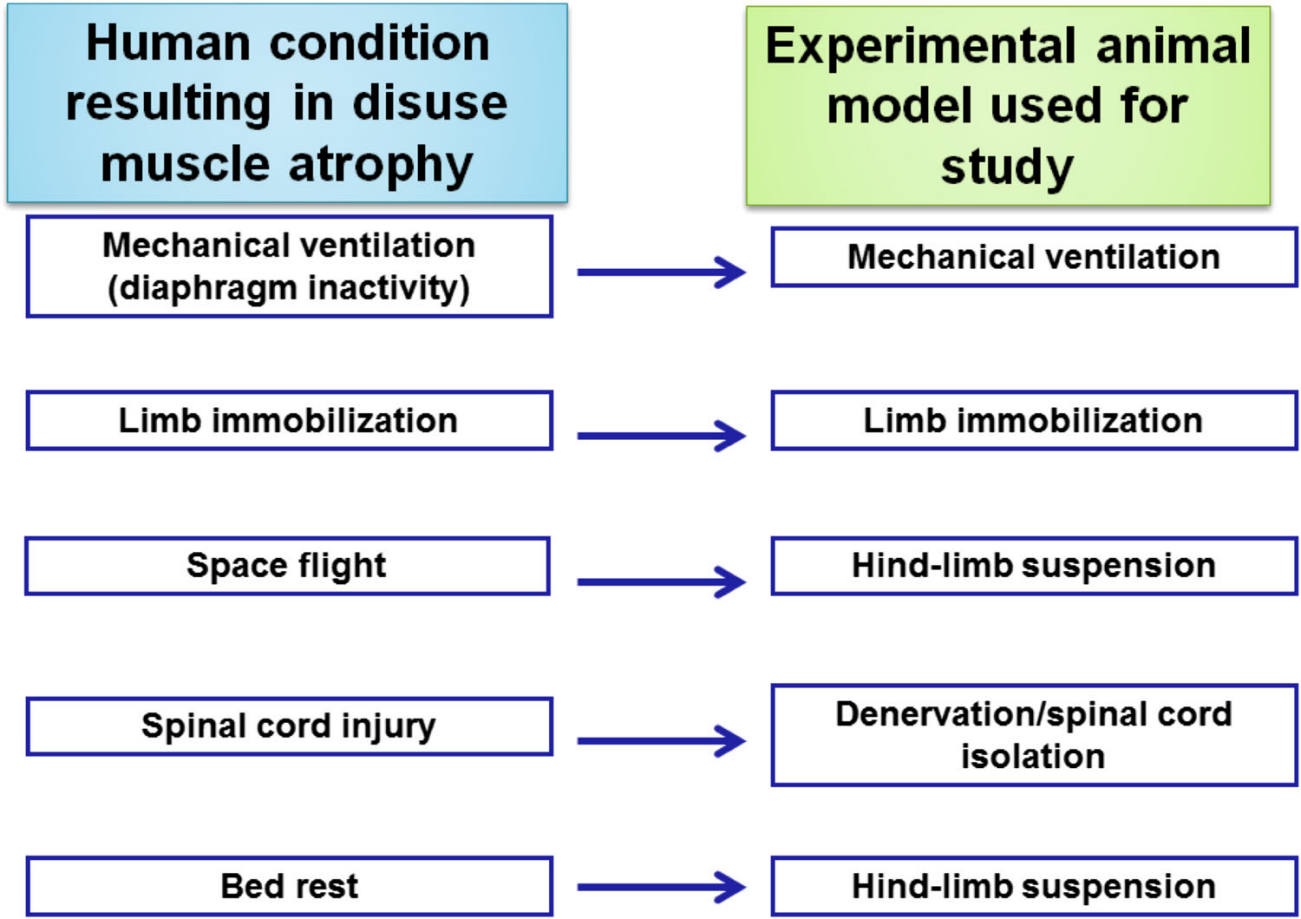
Illustration of several human conditions that promote inactivity-induced skeletal muscle atrophy, along with the corresponding animal model that is commonly used to study each condition

Because of both ethical considerations and the complexities associated with studying the mechanisms responsible for disuse muscle atrophy in humans, animal models are commonly used to study the cellular mechanisms responsible for muscle atrophy. In this regard, several animal models are commonly used to simulate the various types of human disuse muscle atrophy. Specifically, several conditions can result in disuse muscle atrophy in humans: (1) prolonged mechanical ventilation, resulting in inactivity of inspiratory muscles; (2) broken bone that requires limb immobilization (i.e. casting), and the resulting muscle inactivity; (3) space flight, resulting in unloading of skeletal muscles; (4) spinal cord injury; and (5) prolonged bed rest. For each of these human conditions that result in muscle atrophy, a corresponding rodent model exists (Fig. 1). For example, a tail suspension technique to unload the hindlimb muscles of rodents is frequently utilized to replicate the human muscle atrophy that occurs during space flight or prolonged bed rest. Also, experimental manipulations such as denervation or spinal cord isolation have been employed to investigate the impact of prolonged muscle inactivity on rodent skeletal muscle structure and function.

In addition to the numerous studies performed on limb skeletal muscles, a growing number of reports have examined the impact of reduced contractile activity on respiratory muscle fibre size and function in both humans and animals. A common and clinically relevant experimental model to study inactivity-induced inspiratory muscle atrophy is mechanical ventilation. Specifically, full-support mechanical ventilation is a clinically important intervention, which can be life-saving in patients with respiratory failure. However, during full-support mechanical ventilation, the ventilator delivers all of the breaths and the patient’s respiratory muscles are inactive. Both human and animal studies have demonstrated that prolonged mechanical ventilation results in extremely rapid inspiratory muscle (i.e. diaphragm) atrophy. Indeed, as few as 18 h of mechanical ventilation can result in significant diaphragmatic atrophy (e.g. >15 % reduction in fibre cross-sectional area) in both humans and rodents [9, 10]. This rate of disuse muscle atrophy is unique, as a comparable level of disuse muscle atrophy in limb skeletal muscles would require at least 96 h of muscle inactivity to achieve [11, 12].

## Disuse Muscle Atrophy: the Big Picture

**Fig. 2:**
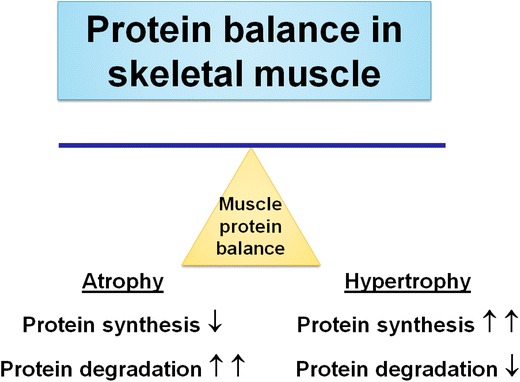
Conservation of skeletal muscle mass depends on the balance between the rates of protein synthesis and degradation. An increase in the rate of protein synthesis relative to the rate of protein breakdown results in muscle hypertrophy. Conversely, an increase in the rate of protein breakdown relative to the rate of protein synthesis results in a net loss of muscle protein, and fibre atrophy occurs

The control of skeletal muscle fibre size is determined by balancing the rates of protein synthesis and degradation (Fig. 2). Animal studies have established that inactivity-induced skeletal muscle atrophy occurs because of both increased proteolysis and decreased muscle protein synthesis [11, 13]. For example, muscle protein synthesis declines within 6 h following the initiation of muscle inactivity [11, 13]. Further, animal studies have revealed that disuse muscle atrophy is also due to large increases in muscle protein breakdown [10, 11].

## How Do ROS Promote Disuse Muscle Atrophy?

The notion that increased ROS production and disturbances in redox signalling play a significant role in the promotion of disuse muscle atrophy was proposed over 20 years ago [14]. Nonetheless, this view did not receive substantial scientific investigation until recent years. In the following sections, the cellular locations responsible for ROS production in inactive skeletal muscle are discussed, and evidence supporting the position that inactivity-induced oxidative stress plays a key role in the regulation of both muscle protein synthesis and degradation is presented.

### Prolonged Skeletal Muscle Inactivity Increases ROS Production

The first evidence that contracting skeletal muscles produce ROS was reported over 30 years ago by Kelvin Davies and colleagues [15]. Since that seminal report, many studies have confirmed that resting skeletal muscles produce low levels of ROS and that the initiation of contractile activity results in marked increases in ROS production in the active fibres [16–20]. Paradoxically, it was reported in 1991 that prolonged skeletal muscle inactivity due to immobilization resulted in chronic increases in ROS production and oxidative damage in the inactive muscle fibres [14]. This ground-breaking discovery has been confirmed by many studies over two decades (reviewed in references [3–6]).

Although a detailed understanding of why prolonged inactivity results in increased ROS production in inactive skeletal muscle remains elusive, a growing number of studies have provided insight into this interesting biological phenomenon. Although both xanthine oxidase and nicotinamide adenine dinucleotide phosphate (NADPH) oxidase contribute to inactivity-induced ROS production in skeletal muscle [21, 22], mitochondria appear to be the dominant site of ROS production in inactive skeletal muscles [23–26]. For example, mitochondria isolated from diaphragm muscle of mechanically ventilated animals released ~40 % more ROS during state 4 respiration than mitochondria obtained from the diaphragm of control animals [23]. Further, treatment of animals with a mitochondrial-targeted antioxidant prevented inactivity-induced oxidative stress in the diaphragm of animals exposed to prolonged mechanical ventilation [26]. Similar results have been reported in immobilized limb muscles [24, 25]. The mechanisms responsible for this inactivity-induced increase in mitochondrial ROS production remain unknown (see reference [5] for a review).

### Oxidative Stress Can Depress Protein Synthesis

Emerging evidence indicates that exposure of cells to high levels of ROS can depress protein synthesis. This topic has been addressed in detail in a previous review [5]; therefore, only a brief synopsis of these findings is presented here. Briefly, protein synthesis in cells is accomplished by a highly structured scheme of signalling pathways, which culminate in the translation of messenger RNA (mRNA) into a specific protein. The rate of protein synthesis is primarily controlled by the efficiency of translation, which is regulated at the level of initiation [27]. Mounting evidence indicates that oxidants depress protein synthesis by hindering mRNA translation at the level of initiation (reviewed in reference [4]). For example, exposure of cardiac myocytes to oxidative stress [i.e. exposure to hydrogen peroxide (H_2_O_2_)] inhibits global protein synthesis by ~90 % [28]. It follows that the decrease in protein synthesis that occurs in skeletal muscle exposed to prolonged skeletal muscle inactivity could be mechanistically linked to increased production of ROS within the inactive muscle fibres.

### Oxidative Stress Increases Proteolysis in Skeletal Muscle

It has been postulated that increased skeletal muscle inactivity-induced increases in ROS production and the resulting oxidative stress accelerate muscle protein breakdown in three different ways. First, oxidative stress promotes the expression of proteins involved in at least three proteolytic systems, including autophagy, calpain and the ubiquitin–proteasome system of proteolysis. Second, inactivity-induced oxidative stress in skeletal muscle results in the activation of two important proteases, calpain and caspase-3. Finally, increased ROS production in muscle fibres can also promote proteolysis by oxidative modification of myofibrillar proteins, which enhances their susceptibility to proteolytic processing. A brief summary of these connections between oxidative stress and proteolysis is presented in the following sections.

#### Oxidative Stress Increases Synthesis of Proteolytic Proteins

The major proteolytic systems found in skeletal muscle can be categorized into four groups: (1) autophagy (i.e. lysosomal proteases); (2) the ubiquitin–proteasome system; (3) calpains; and (4) caspase-3. Growing evidence reveals that cellular oxidative stress can increase the expression of key proteins involved in autophagy and the ubiquitin–proteasome system, and can increase expression of both calpain 1 and calpain 2. Specific details of these processes are highlighted in the following sections.

*ROS Increase Expression of Key Autophagy Proteins* Autophagy is a highly regulated proteolytic pathway for the degradation of non-myofibril cytosolic proteins and organelles [29]. During autophagy, cytosolic components (i.e. proteins and organelles) are sequestered into vesicles called autophagosomes. After formation, these autophagosomes fuse to lysosomes and the cytosolic constituents are degraded by lysosomal proteases (i.e. cathepsins) [30]. Although it has been established that several lysosomal proteases (i.e. cathepsins B, D and L) are activated in skeletal muscle undergoing disuse atrophy [31], the role that the autophagic proteolytic system plays in muscle atrophy has received limited research attention. Nonetheless, emerging studies have revealed that accelerated autophagy contributes to skeletal muscle atrophy in response to both fasting and denervation [32, 33]. Furthermore, a recent study has demonstrated that autophagosomes are formed in diaphragm muscle during prolonged mechanical ventilation, suggesting that autophagy contributes to ventilator-induced protein breakdown in the diaphragm [34].

Emerging evidence indicates that oxidative stress increases the expression of autophagy genes in skeletal muscles. Indeed, a recent study has revealed that increased cellular ROS production promotes the expression of the autophagy-related beclin 1 and cathepsin L genes in cultured cells [35]. Importantly, another report has indicated that inactivity-induced oxidative stress also promotes the expression of autophagy-related proteins in human skeletal muscle [34]. These findings have been confirmed in rodent locomotor muscles exposed to prolonged immobilization [24]. Specifically, prevention of inactivity-induced increases in mitochondrial ROS emission in hindlimb muscles prevents the expression of cathepsin L [24]. Together, these results suggest that oxidative stress increases gene expression of selected autophagy-related genes, which have the potential to increase the rate of autophagy-mediated protein breakdown in cells.

*Oxidative Stress Increases Expression of Proteins Required for the Ubiquitin*–*Proteasome System* The ubiquitin–proteasome system comprises the total proteasome complex (26S), which includes a core proteasome subunit (20S) combined with a regulatory complex (19S) attached at the end of the 20S proteasome core [36, 37]. The 26S proteasome degrades ubiquitinated proteins only. Hence, the 26S proteasome degradation pathway is active when ubiquitin binds to protein substrates and labels these molecules for breakdown. The binding of ubiquitin to protein substrates is a three-step process, which requires the participation of three families of ubiquitin-activating enzymes [4]. In this regard, evidence indicates that the ubiquitin-conjugating enzyme E2_14k_ is an important regulator of ubiquitin–protein conjugation in skeletal muscle [1]. Further, several skeletal muscle-specific ubiquitin E3 ligases (e.g. atrogin-1 and muscle ring finger-1) exist, and these proteins play important roles in skeletal muscle atrophy [38, 39].

Abundant evidence confirms that oxidative stress promotes increased gene expression of proteins involved in the ubiquitin–proteasome system. For instance, in vitro experiments have revealed that exposure of C2C12 myotubes to H_2_O_2_ increases the expression of specific ubiquitin-activating enzymes that contribute to muscle protein breakdown, including E2_14k_, atrogin-1 and muscle ring finger-1 [1, 40]. Similarly, in vivo experiments have revealed that oxidative stress augments the expression of atrogin-1 and muscle ring finger-1 in rodent skeletal muscles [24, 26]. Together, these results verify that ROS-induced oxidative stress promotes the expression of key components of the ubiquitin–proteasome system of proteolysis in skeletal muscle.

*Oxidative Stress Increases Calpain Expression* Calpains are Ca^2+^-dependent cysteine proteases and are located in all mammalian cells [41]. While several calpain isoforms exist, the two best characterized calpains located in skeletal muscle are calpains 1 and 2 [41]. Active calpains promote the release of sarcomeric proteins by cleaving cytoskeletal proteins (e.g. titin and nebulin) that anchor contractile elements [41–43]. Further, calpain can break down selected kinases and phosphatases, and can also degrade oxidized contractile proteins, such as actin and myosin [41, 44].

Several reports have indicated that oxidative stress increases the expression of calpains in both C2C12 myotubes and human myoblasts. For example, exposure of C2C12 myotubes to H_2_O_2_ increases calpain 1 mRNA levels [40]. Further, exposure of human myoblasts to H_2_O_2_ promotes the expression of both calpain 1 and calpain 2 [45]. Together, these investigations suggest that oxidative stress increases calpain expression in cultured muscle cells. At present, it is unknown if oxidative stress can increase the expression of calpain in skeletal muscle fibres in vivo.

#### Oxidative Stress Increases Protease Activation

As discussed in the previous sections, oxidative stress increases the expression of several important proteolytic proteins. This section presents robust evidence demonstrating that oxidative stress can promote the activation of selected proteases (e.g. calpain and caspase-3) in skeletal muscles.

*Elevated Cellular ROS Production Activates Calpain* Numerous studies have concluded that oxidative stress increases calpain activity in muscle cells. For example, treatment of C2C12 myotubes with H_2_O_2_ activates calpain 1 and stimulates myotube atrophy [40]. Similarly, exposure of human myoblasts to H_2_O_2_ increases the activities of both calpain 1 and calpain 2 [45]. Moreover, prevention of oxidative stress via antioxidants can prevent calpain activation in inactive diaphragm muscle in vivo [26, 46]. Similarly, mitochondrial-targeted antioxidants can prevent calpain activation in immobilized limb muscles [24]. Collectively, these studies have confirmed that increased production of ROS in skeletal muscle promotes the activation of calpain.

The mechanism(s) responsible for ROS-mediated calpain activation appear(s) to be linked to oxidative stress-induced disturbances in calcium homeostasis. The two key factors that regulate calpain activity in cells are cytosolic calcium levels and the concentration of the endogenous calpain inhibitor, calpastatin [41]. Specifically, calpain can be activated by a sustained elevation in cytosolic free calcium and/or a decrease in cytosolic levels of calpastatin [41]. During prolonged periods of muscle inactivity, it is established that muscle inactivity is accompanied by elevated cytosolic calcium levels and increased calpain activation [47]. Although the mechanism responsible for this disuse-induced increase in cellular calcium remains under investigation, it is possible that increased ROS production plays a key role in this event [48]. A potential connection between oxidative stress and increased cytosolic calcium is ROS-driven formation of reactive aldehydes (i.e. 4-hydroxy-2,3-trans-nonenal), which can impede plasma membrane Ca^+2^ ATPase activity [49]. Logically, a decline in membrane Ca^+2^ ATPase activity would hinder Ca^+2^ removal from the cell, resulting in increased cytosolic Ca^+2^. It is also possible that oxidation of the ryanodine receptor can increase Ca^+2^ leakage from the sarcoplasmic reticulum, resulting in increased cytosolic Ca^+2^ levels [50]. Nonetheless, it is not clear which of these mechanisms is responsible for inactivity-mediated calcium overload within skeletal muscle, and this subject remains an active area of research.

*Oxidative Stress Promotes Caspase-3 Activation* Research has revealed that the activation of caspase-3 contributes to skeletal muscle protein degradation and fibre atrophy [51–53]. Specifically, active caspase-3 results in the degradation of actomyosin complexes, and inhibition of caspase-3 activity suppresses the overall rate of proteolysis in diabetes-mediated cachexia and disuse-induced muscle atrophy [51–53].

Numerous studies have confirmed that oxidative stress activates caspase-3 in skeletal muscle. For instance, exposing C2C12 myotubes to H_2_O_2_ activates caspase-3 [54]. Further, several studies have confirmed that prevention of inactivity-induced oxidative stress in skeletal muscles prevents caspase-3 activation [24, 26, 46].

Control of caspase-3 activity in cells is complex and involves numerous signalling pathways. Inactivity-induced caspase-3 activation in skeletal muscle can occur by activation of caspase-12 via a calcium release pathway and/or activation of caspase-9 via a mitochondrial signalling pathway [7]. A potential interaction between these caspase-3 activation pathways is that both signalling pathways can be activated by ROS [3, 55]. Finally, note that caspase-3 can also be activated by calpain activation via a cross-talk mechanism between these two proteases [51, 56]. Regardless of which pathway is responsible for inactivity-induced caspase-3 activation in skeletal muscle, it is apparent that ROS can promote caspase-3 activation.

#### Protein Oxidation Accelerates Proteolysis

Another link between oxidative stress and increased protein turnover is that oxidation of skeletal muscle proteins increases their vulnerability to proteolytic breakdown. Indeed, Davies and Goldberg were the first to demonstrate that ROS accelerate proteolysis [57]. This early work has been confirmed, and it is now clear that oxidized proteins are swiftly degraded by several proteases, including the ubiquitin–proteasome system [36, 37, 44]. Further, evidence has revealed that oxidation increases the susceptibility of skeletal muscle myofibrillar proteins to degradation by both calpains and caspase-3. Indeed, oxidation of sarcomeric proteins (e.g. myosin heavy chain, α-actinin, actin and troponin I) increases their breakdown by both calpain and caspase-3 in a dose-dependent manner [44].

The connection between high cellular levels of ROS and accelerated protein breakdown is due, in part, to the fact that oxidation of muscle proteins results in unfolding of the affected proteins, resulting in enhanced susceptibility to proteolysis [57]—that is, oxidative modification of a protein results in a change in the molecular structure such that the formerly protected peptide bonds are now exposed to enzymatic breakdown [15, 44].

### Summary: How Do ROS Promote Muscle Atrophy?

**Fig. 3:**
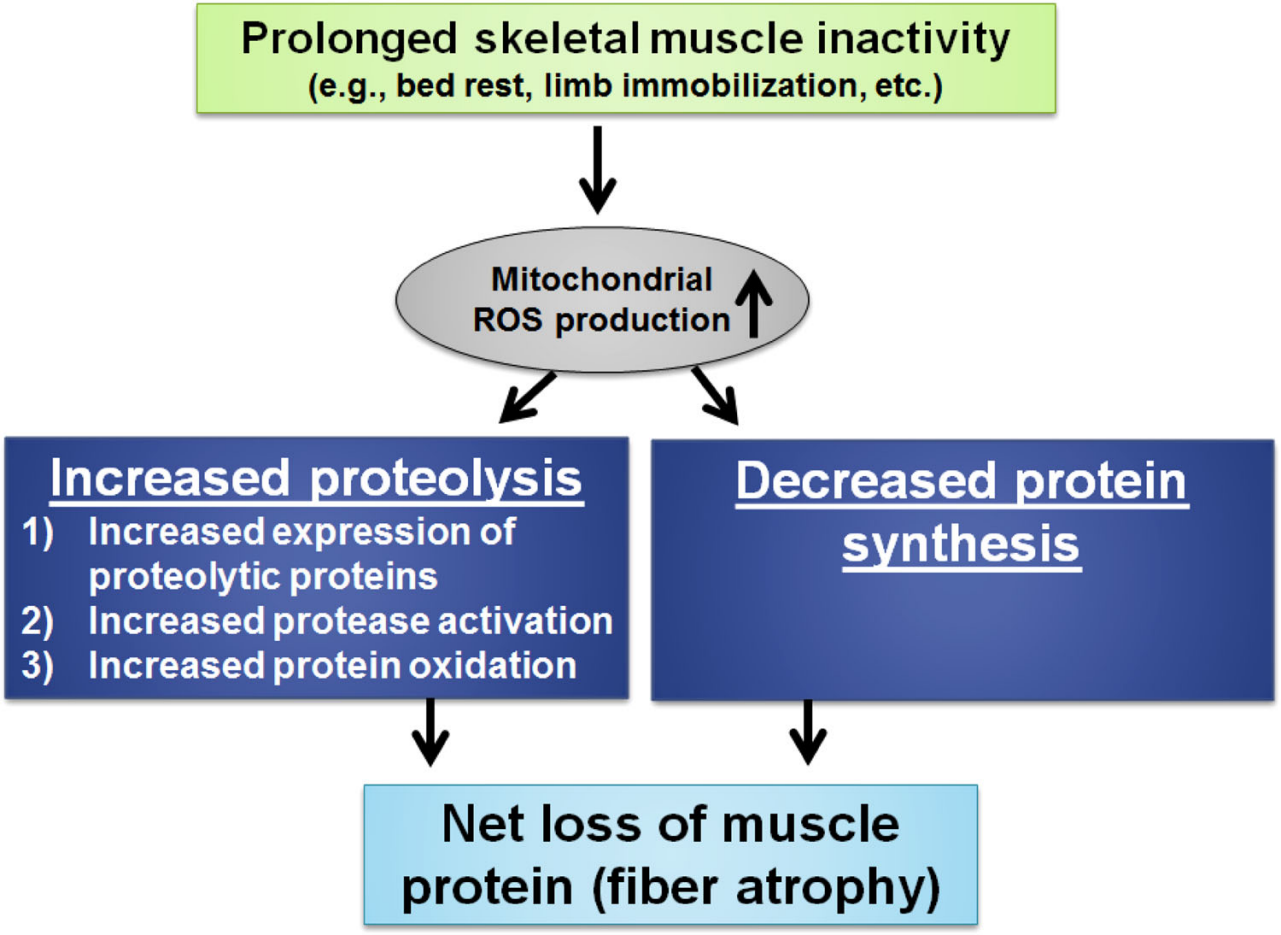
Steps leading from oxidative stress to muscle fibre atrophy. Inactivity-induced oxidative stress can promote muscle protein breakdown in three major ways: (1) oxidative stress increases gene expression of key proteolytic proteins; (2) cellular oxidative stress can activate selected proteases (i.e. calpain and caspase-3); and (3) oxidants can oxidize myofibrillar proteins and enhance their susceptibility to proteolytic processing. Further, oxidative stress can depress muscle protein synthesis. Collectively, this increased proteolysis and decreased muscle protein synthesis result in a net loss of muscle protein and, consequently, fibre atrophy. *ROS* reactive oxygen species

Prolonged muscle inactivity results in increased mitochondrial ROS production and, consequently, disturbed redox signalling (i.e. oxidative stress) in the inactive skeletal muscle fibres. This inactivity-induced oxidative stress results in increased proteolysis in muscle fibres, which is due to increased expression of key proteolytic proteins, augmented protease activation and increased oxidation of skeletal muscle proteins. Further, oxidant stress has the potential to depress muscle protein synthesis. Collectively, the ROS-induced increase in proteolysis and decrease in protein synthesis results in a net loss of muscle protein (Fig. 3).

## Can Treatment with Antioxidants Prevent Disuse Muscle Atrophy?

The previous sections discussed the factors that explain the biological connection between increased ROS production and inactivity-induced skeletal muscle atrophy. A frequent experimental approach to determine cause and effect between increased ROS production and disuse muscle atrophy is the treatment of animals with antioxidants to prevent inactivity-induced oxidative stress in the inactive muscles. Using this approach, evidence exists both for and against the notion that antioxidants can prevent disuse muscle atrophy. Most of these studies have used an individual antioxidant. In the following sections, the efficacy of specific antioxidants to protect against disuse muscle atrophy is examined. The discussion focuses on those antioxidants that have received significant experimental attention.

### Vitamin E/Vitamin E Analogues and Disuse Muscle Atrophy

Both vitamin E and vitamin E analogues have been widely investigated as antioxidant interventions to protect against disuse muscle atrophy [12, 14, 46, 58–60]. Vitamin E is one of the most widely distributed antioxidants in nature and is the major antioxidant found in cell membranes. The generic term ‘vitamin E’ refers to eight structural isomers of tocopherols and tocotrienols [61]. Among these molecules, α-tocopherol (the all-rac form) is the best known and possesses the highest antioxidant capacity [61]. In addition to its antioxidant activity, vitamin E is known to promote gene expression of selected muscle proteins [61].

In regard to vitamin E and muscle atrophy, Kondo et al. [14] provided the first investigation demonstrating that administration of vitamin E to rats protected against immobilization-induced hindlimb muscle atrophy. Since that early study, several other reports have concluded that vitamin E can completely or partially protect rodents against muscle atrophy induced by hindlimb unloading, immobilization or denervation [59, 60, 62, 63].

Although studies have reported that vitamin E can blunt disuse muscle atrophy, the mechanisms behind this protection remain unclear. However, a recent study has suggested that the protective effect of vitamin E against disuse muscle atrophy could be due to modulation of muscle proteolysis-related genes, rather than its antioxidant function [60]. For example, treatment of animals with high levels of vitamin E (60 mg/kg body mass, twice weekly) increased the expression of heat shock protein 72 and decreased the expression of several proteases, including calpain and caspase-3, in inactive skeletal muscles [60]. In theory, both of these changes in gene expression could provide protection against disuse muscle atrophy [56, 64–66]. Therefore, it is possible that although vitamin E protects against disuse muscle atrophy, this protection is achieved by alterations in gene expression and not necessarily the antioxidant function of vitamin E [60]. Clearly, additional research is required to determine the precise mechanism(s) responsible for vitamin E-mediated protection against disuse muscle atrophy.

Trolox (6-hydroxy-2,5,7,8-tetramethylchromane-2-carboxylic acid) is a cell-permeable and water-soluble analogue of vitamin E [67]. Like vitamin E, Trolox has antioxidant properties that are derived from direct scavenging of H_2_O_2_ and other ROS [67]. Several investigations have demonstrated that treatment of animals with Trolox (i.e. an intravenous infusion of 4 mg/kg/h) protects the diaphragm against ventilator-induced diaphragmatic atrophy [2, 12, 46, 58]. The mechanism responsible for this protection appears to be, at least in part, related to the fact that oxidative stress is required to activate both calpain and caspase-3 in the diaphragm during prolonged mechanical ventilation [26, 46].

In contrast to the findings that Trolox protects against ventilator-induced diaphragmatic atrophy, two studies have concluded that Trolox supplementation does not protect against inactivity-induced limb muscle wasting. Specifically, these studies demonstrated that treatment of mice with Trolox did not protect against hindlimb unloading-induced muscle atrophy [68, 69]. The reason(s) for these divergent findings across the Trolox studies are unclear but could be related to the duration of muscle inactivity and/or the dosage of Trolox that was used in the experiments.

### *N*-Acetylcysteine and Disuse Muscle Atrophy

*N*-Acetylcysteine is a small molecule comprising cysteine with an acetyl group attached to the nitrogen atom. *N*-acetylcysteine is widely used clinically as a mucolytic agent and as a treatment for paracetamol (acetaminophen) overdose. Further, *N*-acetylcysteine is also sold as a nutritional supplement, is a direct scavenger of ROS [70] and can provide cysteine for glutathione synthesis (i.e. glutathione is an important non-enzymatic antioxidant) [71]. To date, only two studies have investigated the ability of *N*-acetylcysteine to protect against disuse muscle atrophy. The first study concluded that although treatment of animals with *N*-acetylcysteine in the diet prevented the increase in nuclear factor (NF)-kappaB activity induced by hindlimb suspension, *N*-acetylcysteine treatment did not protect against inactivity-induced muscle atrophy [72]. In contrast, another investigation demonstrated that treatment of animals with *N*-acetylcysteine (150 mg/kg intravenously) prevented mechanical ventilator-induced oxidative stress, averted the activation of both calpain and caspase-3, and protected the diaphragm against disuse-induced diaphragm fibre atrophy [70]. It is difficult to determine if these divergent results are due to a disparity between studies in the plasma levels of *N*-acetylcysteine resulting from the different routes of drug delivery (i.e. dietary intake versus intravenous infusion), and additional research is required to determine whether *N*-acetylcysteine has the potential to be an effective agent against disuse-induced muscle atrophy.

### Mitochondrial-Targeted Antioxidants and Disuse Muscle Atrophy

As discussed earlier, mitochondria appear to be the dominant site of ROS production in skeletal muscles during prolonged periods of inactivity [23–26]. Therefore, in theory, a mitochondrial-targeted antioxidant could prevent inactivity-induced oxidative stress and protect muscles against disuse atrophy. In this regard, three reports have concluded that a mitochondrial-targeted antioxidant can protect both limb and respiratory muscles against inactivity-induced atrophy. Specifically, SS-31 (d-Arg-2′6′dimethylTyr-Lys-Phe-NH_2_) is a synthetic aromatic cationic tetrapeptide that selectively targets and concentrates in the inner mitochondrial membrane [73]. Importantly, the mitochondrial targeting of SS-31 provides selective scavenging of mitochondrial ROS. Numerous studies in isolated mitochondria, cultured cells and animal models have shown that SS-31 can selectively scavenge mitochondrial ROS and protect mitochondrial function during periods of increased ROS production [25, 26, 74, 75]. The first report documenting the ability of a mitochondrial-targeted antioxidant to protect against disuse muscle atrophy revealed that treatment of animals with SS-31 protects the rat diaphragm against the disuse muscle atrophy that occurs during prolonged mechanical ventilation [26]. Subsequently, two additional investigations have concluded that treatment of both rats and mice with SS-31 protects against immobilization-induced atrophy in hindlimb muscles [24, 25]. The mechanism of protection against atrophy by this specific mitochondrial-targeted antioxidant appears to be protection against inactivity-induced oxidative stress and prevention of protease activation (e.g. caspase-3 and calpain) in the muscle [24, 26].

### Miscellaneous Antioxidants/Antioxidant Cocktails and Disuse Muscle Atrophy

While vitamin E, Trolox and mitochondrial-targeted antioxidants have received significant investigative attention, a limited number of studies have examined the impact of other antioxidants on disuse muscle atrophy. For example, only two studies have investigated the effect of curcumin (an antioxidant found in the spice turmeric) on disuse muscle atrophy. Unfortunately, these studies arrived at divergent conclusions regarding the ability of curcumin to protect against disuse muscle atrophy. Specifically, one report concluded that curcumin (a 1 % dose in the diet) did not protect against hindlimb unloading-induced muscle atrophy [72]. In contrast, the second report concluded that treatment with curcumin (600 mg/kg given intraperitoneally three times daily) protected the diaphragm against ventilator-induced muscle wasting [76]. Unfortunately, it is unclear if these divergent findings are due to the varying routes of curcumin administration (i.e. dietary intake versus intraperitoneal injection) resulting in markedly different levels of circulating curcumin between the two investigations. Clearly, additional experiments are required to resolve whether curcumin has the potential to protect against disuse muscle atrophy.

Further, a new report has suggested that treatment of mice with beta-carotene (a lipid soluble antioxidant) can protect against denervation-induced muscle atrophy [77]. This is an interesting new finding, and additional studies are warranted to determine if beta-carotene is protective against other forms of disuse muscle atrophy.

Finally, one study has investigated the effects of a complex antioxidant cocktail on protection against hindlimb unloading-induced muscle atrophy in rodents [78]. This experiment treated animals with an antioxidant cocktail (including vitamin E, vitamin C and beta-carotene) contained in the diet. Although this form of antioxidant supplementation resulted in increased antioxidant capacity within the hindlimb muscles of the treated animals, it did not protect against hindlimb unloading-induced muscle atrophy [78]. These results clearly illustrate the concept that not all antioxidant treatments are successful in preventing disuse muscle atrophy.

## Summary and Conclusions

**Fig. 4:**
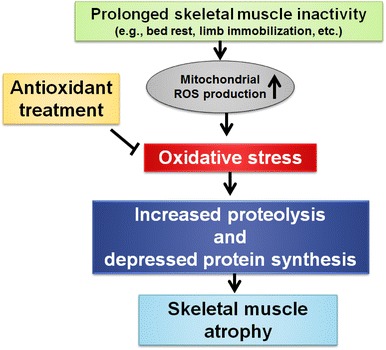
Illustration of the potential role that antioxidants can play in protection against disuse muscle atrophy. Specifically, prolonged skeletal muscle inactivity leads to increased mitochondrial reactive oxygen species (ROS) production and oxidative stress in the inactive muscle fibres. This increased ROS production and oxidative stress can promote decreased protein synthesis and promote proteolysis in the muscle, leading to skeletal muscle atrophy. In theory, treatment with selected antioxidants can block disuse-induced oxidative stress and protect muscle fibres against disuse muscle atrophy

It is well known that disuse skeletal muscle atrophy occurs during prolonged bed rest, during limb immobilization and as a result of prolonged mechanical ventilation. Several lines of evidence couple increased production of ROS to disuse muscle atrophy via ROS-mediated increases in proteolysis. Further, it is also possible that oxidative stress can depress muscle protein synthesis, which is another contributory factor to the loss of muscle protein and fibre atrophy that occurs during prolonged inactivity. Developing an effective countermeasure to protect against inactivity-induced muscle atrophy is important. In this regard, prevention of inactivity-induced muscle atrophy requires an intervention that can maintain protein synthesis and/or decrease proteolysis in muscles exposed to prolonged periods of inactivity. Therefore, since antioxidants can prevent inactivity-induced oxidative stress in skeletal muscles, treatment of animals with antioxidants could potentially maintain protein synthesis and prevent accelerated proteolysis (Fig. 4). Indeed, several studies have suggested that selected antioxidants (e.g. vitamin E, Trolox and mitochondrial-targeted antioxidants) have the potential to decrease inactivity-induced muscle atrophy of both limb and respiratory muscles. Nonetheless, at present, the use of antioxidants as a therapeutic intervention to protect athletes and patient populations against disuse muscle atrophy is not widely accepted. Therefore, additional research is required to completely establish that antioxidant treatments are both safe and effective in protecting against inactivity-induced muscle atrophy.
